# Nutritional Aspects of Eggs for a Healthy and Sustainable Consumption: A Narrative Review

**DOI:** 10.1002/fsn3.70285

**Published:** 2025-09-05

**Authors:** Irene Caffa, Elisa Proietti, Federica Turrini, Consuelo Borgarelli, Maria Regina Ferrando, Elena Formisano, Lenycia de Cassya Lopes Neri, Daniela Martini, Donato Angelino, Anna Tagliabue, Livia Pisciotta

**Affiliations:** ^1^ Department of Internal Medicine and Medical Specialties University of Genoa Genoa Italy; ^2^ Ospedale Policlinico San Martino IRCCS Genoa Italy; ^3^ Department of Pharmacy University of Genoa Genoa Italy; ^4^ National Center for the Development of New Technologies in Agriculture (Agritech) Napoli Italy; ^5^ Laboratory of Food Education and Sport Nutrition, Department of Public Health, Experimental and Forensic Medicine University of Pavia Pavia Italy; ^6^ Human Nutrition and Eating Disorder Research Center, Department of Public Health, Experimental and Forensic Medicine University of Pavia Pavia Italy; ^7^ Department of Food, Environmental and Nutritional Sciences (DeFENS) Università Degli Studi di Milano Milano Italy; ^8^ Department of Bioscience and Technology for Food, Agriculture and Environment University of Teramo Teramo Italy

**Keywords:** cardiovascular disease, cholesterol, egg composition, egg consumption, sustainability

## Abstract

Despite being recognized as a low‐cost food, rich in proteins and other nutrients, for years eggs have been the subject of controversy regarding a possible negative impact on human health linked to their frequent consumption and their cholesterol content. This narrative review describes the composition of eggs, the properties of individual nutrients, and the impact of their deficiency or excess on human health, and the development of several pathologies. The chemical–physical properties of the proteins and lipids contained in eggs and the environmental impact linked to their production are also considered. In addition to covering fundamental functions for the maintenance of cellular structures and functions, the nutrients present in eggs show in vitro antioxidant, antimicrobial, antihypertensive, anti‐inflammatory, and immunomodulatory functions, which could be fundamental in protecting against pathologies such as tumors or neurodegenerative diseases. Recent clinical studies have also highlighted the lack of correlation between egg consumption and cardiovascular disease. The chemical–physical properties of the proteins and lipids present in eggs also make them fundamental in many food preparations. Eggs are an important source of energy, nutrients, and are particularly useful in the food industry. Egg production, especially in small‐scale poultry systems, is also environmentally sustainable.

## Graphical abstract

In this review we have described the structural and bromatological composition of the different varieties of eggs available in the market, analyzing in detail the biological role of the various nutrients present in eggs and their involvement in health and disease. We also covered the recommendations on egg consumption depending on consumers' age given by different Italian and international associations/organizations. We have extensively described the functional properties of eggs and finally we focused on the sustainability linked to the environmental footprint of the agri‐food systems.

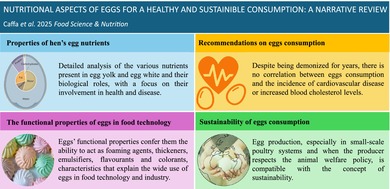

## 1 Introduction

Hen's eggs have long been of great interest in the field of nutrition, being considered the most affordable source of animal protein and boasting a relatively low‐calorie content of about 130 kcal/100 g (Gnagnarella et al. 2022). At the same time, due to the high cholesterol content in whole eggs (358 mg per 100 g), eggs have long been excluded from medicals' and nutritionists' recommendations, being considered detrimental to cardiometabolic health (Mohseni et al. 2023). However, it is now clear that dietary guidelines should focus on improving the overall quality of the diet to promote cardiovascular and overall health, rather than strongly criticizing individual foods, such as eggs. Among various dietary models, the Mediterranean Diet and the Dietary Approaches to Stop Hypertension (DASH) diet appear to be healthy and heart‐protective eating styles (Mach et al. 2020). They emphasize the consumption of vegetables, fruits, whole foods, healthy protein sources, vegetable oils, and minimally processed foods. Eggs are not excluded by these dietary models; instead, they are incorporated as a protein option in both (McNamara 2015; Kim and Campbell 2018; Lichtenstein et al. 2021). To discuss the topic, a narrative review was conducted by an *ad hoc* working group of the Italian Society of Human Nutrition.

## 2 Structural and Bromatological Composition of Hen's Eggs

**FIGURE 1:**
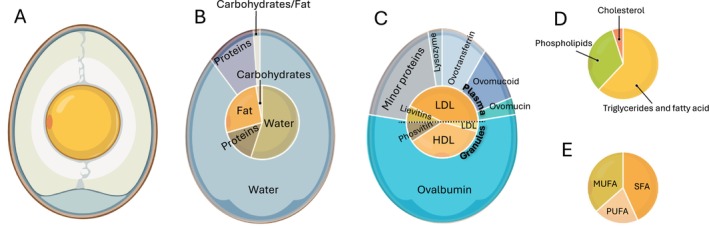
Composition of hen's eggs. (A) Dimension and white/yolk ratio. (B) Macronutrients composition of white and yolk. (C) Major protein composition of white and yolk. (D) Lipid composition of egg yolk. (E) Fatty acid profile of egg yolk. MUFA, monounsaturated fatty acids; PUFA, polyunsaturated fatty acid; SFA, saturated fatty acids.

According to their weight, hen's eggs available on the market are classified into four groups: small (S), medium (M), large (L), and extra‐large eggs (XL) (Table 1). Although S and XL eggs are commonly used in egg‐based preparations, M and L eggs are the most required by customers and, therefore, the most sold worldwide (FAO 2008). Considering a medium hen's egg weighing 55 g, including the shell, which weighs 5 g, the other components of white and yolk are about 35 g and 15 g, respectively (Figure 1A) (CREA, n.d.). The white/yolk ratio and the size of the eggs could be influenced by hen's age: initially, a hen's egg contains 23% yolk, with this percentage increasing to over 28% towards the end of the production cycle (Gautron et al. 2022), whereas the weight of the whole egg varies between 50 g (for younger hens) and 80 g (for older ones) (Rehault‐Godbert et al. 2019). A first distinction on eggs composition has to be made between macronutrients and micronutrients:

– macronutrients, which include water, proteins, lipids, and carbohydrates, have a relative content that remains overall quite similar, depending mainly on the white/yolk ratio (Table 2, Figure 1B);
– micronutrients, which include vitamins, choline, minerals, and trace elements, have a more variable composition and can be influenced by various factors, such as oviparous feed or age (Table 2) (Rehault‐Godbert et al. 2019).

**TABLE 1:** Classification of hen eggs according to their weight.

Weight of hen's eggs	
XL — extra large	≥ 73 g
L — large	63 g ≤ weight < 73 g
M—medium	53 g ≤ weight < 63 g
S — small	< 53 g

**TABLE 2:** Composition of white, yolk and whole hen's eggs (100 g), according to Banca Dati di Composizione degli Alimenti per Studi Epidemiologici in Italia (BDA).

	White (100 g)	Yolk (100 g)	WHOLE EGG (100 g)	
Energy	43 kcal	325 kcal	128 kcal	
Water	87.7	53.2	77.1	Macronutrients (g)
Proteins	10.9	15.9	12.5	
Lipids	0.17	29.1	8.7	
Cholesterol	0	1.33	0.371	
Carbohydrates	Traces	Traces	Traces	
Calcium	7	116	48	Minerals (mg)
Sodium	179	43	137	
Potassium	135	90	133	
Phosphorus	15	586	210	
Zinc	0.3	2.1	1.2	
Magnesium	12	14	13	
Selenium	0.003	0.01	0.006	
Iron	0.1	4.9	1.5	
Copper	0.04	0.2	0.06	
Chlorine	170	140	160	
Manganese	Traces	0.1	Traces	
Sulfur	180	170	180	
Iodine	0.003	0.140	0.053	
Vitamin A	0	1.4	0.4	Vitamins (mg)
Thiamin (Vitamin B1)	20 μg	270 μg	90 μg	
Riboflavin (Vitamin B2)	0.27	0.35	0.3	
Niacin (Vitamin B3)	0.1	0.1	0.1	
Pantothenic acid (Vitamin B5)	0.3	4.6	1.77	
Vitamin B6	20 μg	0.3	0.12	
Biotin (Vitamin B8)	7 μg	50 μg	20 μg	
Folate (Vitamin B9)	13 μg	130 μg	50 μg	
Colabamin (Vitamin B12)	1 μg	7 μg	3 μg	
Vitamin D	0	5 μg	2 μg	
Alpha‐tocopherol (Vitamin E)	0	3.1	1.1	
Choline	1	680	285	

## 3 Properties of Hen's Egg Nutrients

### 3.1 Proteins

With an average content of 12.5 g per 100 g of whole raw fresh product, eggs constitute one of the best high‐quality and bioavailable protein sources, containing all nine essential amino acids and providing a low intake of calories and saturated fats compared to other high‐quality protein sources (Puglisi and Fernandez 2022). Among the best‐known functions of egg proteins are the improvement of skeletal muscle in adults and the prevention of sarcopenia in the elderly, especially if combined with moderate physical exercise (Myers and Ruxton 2023). Furthermore, various studies have highlighted the satiating power of eggs, probably due to the amino acid composition of its proteins, and their consumption within a meal translates into a reduced caloric intake at subsequent meals, helping to maintain a normal body mass index (BMI) (Myers and Ruxton 2023). In 2023, Sarantidi et al. analyzed the proteome of egg white and yolk, identifying 371 and 428 new proteins, respectively, and integrated these new data with those already present in the literature thus generating the first‐ever egg white and yolk protein atlas dataset (808 different proteins in egg white, 813 in egg yolk, with 229 proteins common in the two parts) (Sarantidi et al. 2023). This paper highlighted that the quantity of proteins in yolk and white is similar (15.9 g and 10.9 g per 100 g respectively), which however changes the most is the typology of proteins and their molecular functions. From in vitro studies, which may not reflect the exact physiological condition following egg consumption, it emerged that egg proteins belong to one of these categories of bioactivity: antioxidant, antimicrobial, antihypertensive, anti‐inflammatory, and immunomodulatory (Sarantidi et al. 2023). Egg white is composed of approximately 90% water and 10% proteins. The most abundant protein is ovalbumin, approximately 54% of total egg white proteins (EWPs), followed by ovomucoid and ovotransferrin representing each one approximately 10%, ovomucin and lysozyme approximately at 3%, and then all the so‐called minor proteins, such as ovoinhibitor, ovoglycoprotein, ovoflavoprotein, ovomacroglobulin, avidin, and cystatin (Abeyrathne et al. 2013) (Figure 1C). Although the function of ovalbumin is not entirely clear, it is hypothesized that it is a storage protein and constitutes an excellent dietary source of amino acids (Rehault‐Godbert et al. 2019). Ovomucoid is the most highly glycosylated protein among EWPs and is considered the dominant food allergen present in egg white (Jarvinen et al. 2007), but allergenic proteins are also contained in yolk. Egg allergy is a common food allergy in children younger than five, but in 50% of cases, this allergy resolves within a few years (Rehault‐Godbert et al. 2019). Ovotransferrin is known to have strong antioxidant, antibacterial, and immunomodulatory properties thanks mainly to its ability to bind iron, necessary for bacterial proliferation (Rathnapala et al. 2021) and it has also been recognized for its anticancer and antihypertensive properties (Puglisi and Fernandez 2022). In addition to being responsible for the gelatinous nature of egg white, ovomucin has proven antioxidant, antiviral, and antibacterial activities (Omana et al. 2010), and the same antimicrobial activity is also shared by lysozyme (Khorshidian et al. 2022), which is also known to protect against inflammatory bowel disease and have an anti‐tumoral effect in animal models (Puglisi and Fernandez 2022). Among the minor proteins, there are protease inhibitors, such as ovoinhibitor and ovomacroglobulin, which can influence the digestibility of eggs (Rehault‐Godbert et al. 2019), and also many proteins with antihypertensive activity (Liao et al. 2019; Miguel and Aleixandre 2006). The yolk is composed of two fractions that contain different proteins: the granular fraction, non‐soluble aggregates that make up 19%–23% of the dry material of the yolk, and the plasma, the liquid part which constitutes 77%–81% of the dry material (Xie et al. 2020). Yolk proteins are distributed equally between plasma and granules, but, as mentioned above, the type of protein changes. Plasma contains 85% of low‐density lipoprotein (LDL) and 15% of lievitins, anti‐inflammatory proteins that are composed of serum albumin (α‐livetin), glycoproteins (β‐livetin) and immunoglobulins (γ‐livetin) (Meram and Wu 2017). Granules contain 73% high‐density lipoproteins (HDL), 9% of LDL (as apolipoprotein B, apovitellenin‐1) and 18% of the antioxidant protein phosvitin (Marcet et al. 2022) (Figure 1C).

### 3.2 Lipids

Unlike proteins, equally distributed between the yolk and white, the egg lipid content (8.7 g per 100 g of whole raw fresh product) is concentrated almost exclusively in the yolk in the form of triglycerides and fatty acids (62%), phospholipids (33%) and cholesterol (5%), and are the key constituents of HDL, LDL, and very‐low‐density lipoprotein (VLDL) (Figure 1D) (Xiao et al. 2020). As previously mentioned, the lipid content varies only depending on the white/yolk ratio. However, the profile of the fatty acids present is more variable and dependent on the oviparous' diet, body fat, and species (Gnagnarella et al. 2022). The quantity of saturated fatty acids (SFA), monounsaturated fatty acids (MUFA) and polyunsaturated fatty acids (PUFA) is 3.17 g, 2.58 g, and 1.26 g per 100 g of whole egg, respectively (Figure 1E) (Rehault‐Godbert et al. 2019). The most abundant SFA are palmitic acid (C16:0), stearic acid (C18:0) and myristic acid (C14:0). Among MUFA and PUFA, the most abundant are oleic acid (C18:1), palmitoleic acid (C16:1), linoleic acid (C18:2), arachidonic acid (C20:4) and docosahexaenoic acid (C22:6) (Tomaszewska et al. 2021). Phospholipids are essential components of cell membranes and play fundamental roles in various physiological processes (Wang and Tontonoz 2019). Among the phospholipids present in the yolk, we find a majority of phosphatidylcholine (78.5%) and phosphatidylethanolamine (17.5%), then in smaller quantities sphingomyelin (2.5%), plasmalogen (0.9%) and phosphatidylinositol (0.6%) (Abeyrathne et al. 2022). The high cholesterol content (almost 300–400 mg per 100 g of whole raw fresh product) has made this food the subject of numerous controversies due to the possible relationship with high levels of plasma cholesterol and the increased risk of developing cardiovascular diseases (CVDs) (Rehault‐Godbert et al. 2019). Although the lack of correlation between the consumption of eggs “per se” and the risk of developing CVDs has been demonstrated, and although international public health associations have removed the advice to limit their consumption from their guidelines, the controversy on the impact of consuming foods high in cholesterol remains, since food with a high content of cholesterol is also typically rich in saturated fats, which are well documented to increase LDL cholesterol (Carter et al. 2023). To overcome these criticisms, studies have been carried out to try to reduce the cholesterol content in eggs, both through technological methods that allow the removal of cholesterol from the finished product and by modifying the diet of farmed hens; however, these approaches do not appear economically sustainable (Sugano and Matsuoka 2021).

### 3.3 Carbohydrates

Eggs are low in carbohydrates; consequently, the content of simple sugars is also rather low: in 100 g of whole egg, it reaches 0.37 g, in the white, 0.34 g per 100 g of product, and in the yolk, 0.18 g per 100 g (Figure 1B and Table 2). Other simple sugars, such as fructose, galactose, and maltose are present in traces (USDA National Nutrient Database for Standard Reference, Legacy Release 2019).

### 3.4 Vitamins

Excluding vitamin C, totally absent in eggs, it is estimated that the consumption of 2 eggs can cover up to 30% of the daily requirement of other vitamins (vitamin A, E, D and group B vitamins). Data from the National Health and Nutrition Examination Survey on over 65,000 subjects demonstrated that egg consumers have significantly higher levels of intake of vitamin A, vitamin D, and vitamin E compared to non‐egg consumers, thus achieving the daily recommended adequate intake levels (Papanikolaou and Fulgoni 2021). In egg white, only B vitamins are found, whereas yolk contains vitamin A, E, D, and group B vitamins (Table 2) (Rehault‐Godbert et al. 2019).

The term vitamin A actually refers to a vast group of fat‐soluble molecules, such as retinol and its biologically active form all‐trans‐retinoic acid (ATRA), retinyl palmitate, and carotenoids, that can't be produced by the human body and need to be introduced through diet. Depending on the type of feeding of the oviparous, variable concentrations of carotenoids can be found in the lipid matrix, which give the typical yellow to orange color of the yolk (Abeyrathne et al. 2022). Vitamin A is involved in various biological processes, such as cellular differentiation, the correct development of bones, the central nervous system and vision, the immune response, and antioxidant functions (Carazo et al. 2021). Lack of vitamin A can cause vision problems and, if the deficiency is severe, can lead to blindness, respiratory problems, skin problems, anemia, and problems in immune responses. In addition to the recognized role of ATRA, used for the treatment of acute promyelocytic leukemia (Carazo et al. 2021), various trials have shown that blood vitamin A levels correlate with a lower risk of tumor development, and also appear to directly inhibit cell proliferation and the ability to invade and therefore form metastases (Kim et al. 2021).

Group B vitamins are necessary for cellular metabolism, DNA synthesis, myelination, and the normal functioning of the central nervous system and for the maturation of red blood cells (Lyon et al. 2020). In obese adolescents, higher vitamin B intake is inversely associated with being metabolically unhealthy obese (MUO) and therefore with a lower risk of developing obesity‐related metabolic comorbidities (B12 has the strongest correlation) (Poursalehi et al. 2022). On the contrary, B vitamins deficiency is linked to the development of several pathologies, such as megaloblastic anemia, hypercellular and dysplastic bone marrow, sarcopenia, frailty, neurodegenerative diseases, and rheumatic diseases (Mathew et al. 2024; Kato et al. 2024). Regarding oncological pathologies, there are conflicting data: in patients with breast cancer, higher blood levels of vitamin B1 and vitamin B5 and lower levels of vitamin B3 were observed, whereas no correlation was observed for other B vitamins (Xie et al. 2023).

Vitamin D is mostly synthesized by our body through the absorption of sunlight, but eggs are one of the few foods rich in vitamin D. By promoting the absorption and maintenance of adequate levels of calcium, vitamin D has a very important role in ossification and in maintaining bone health. Several clinical studies have attempted to confirm the anti‐tumor role of vitamin D observed in animal models. In 2019, the results of the VITAL study on more than 25 thousand subjects were published, but vitamin D supplementation seemed to reduce the incidence of cancer only in subjects with a normal BMI, not in those overweight or obese (Manson et al. 2019). To overcome vitamin D deficiency, estimated to affect 40% of the European population, the feeding of hens with a richer diet in vitamin D or its active metabolite 25‐hydroxyvitamin D has been tested, achieving an increase in vitamin levels in the eggs, but, despite this enriched feed having no toxic effects on hens, the current limit of European legislation of 80 μg vitamin D/kg feed prevents its use (Wei et al. 2023).

Known mostly for its antioxidant function, vitamin E has also hypocholesterolemic properties and plays an important role in the regulation of cell proliferation and cell cycle and in the response to inflammation (Galli et al. 2022). Its deficiency in children can cause growth retardation, whereas in adults it could lead to the onset of nervous system disorders and metabolic problems (Galli et al. 2022). Although in experimental animal models vitamin E has revealed a powerful anti‐tumor effect in various types of tumors, the results on patients have so far given variable results (Yang et al. 2020). With 3.1 mg per 100 g of yolk, not only are eggs a good source of vitamin E, but it has been observed that the consumption of eggs can facilitate the absorption of the vitamin and also from other co‐consumed low‐fat foods, such as a raw vegetable (Kim et al. 2016).

### 3.5 Choline

Choline is an essential nutrient, sometimes associated with group B vitamins, which can be endogenously synthesized by the liver, but it is the phosphatidylcholine contained in the yolk that contributes significantly to the intake of choline in the diet. Choline has important and multiple functions, such as the well‐known role in maintaining the structural integrity of the cell membrane, the transmission of nervous signals after its transformation into the neurotransmitter acetylcholine (Rehault‐Godbert et al. 2019) and the anti‐inflammatory effect, as confirmed by the reduction of plasma markers of inflammation, C‐reactive protein (CRP) and tumor necrosis factor‐α (TNF‐α) induced in healthy adults by choline consumption (Detopoulou et al. 2008). Despite the conflicting data from various studies regarding the correlation between choline intake, BMI, and CVDs, the recent study from Zhou et al. on more than 14,000 multi‐ethnic subjects confirmed that a high choline intake correlates with a lower risk of developing CVDs (even if blood LDL values do not decrease significantly), especially in subjects with BMI < 30 kg/m^2^ (Zhou et al. 2023). In obese individuals, higher choline intake is correlated with being less likely to be MUO and therefore with a lower risk of developing CVDs (Poursalehi et al. 2022). It has recently been demonstrated that choline can reduce insulin, insulin resistance, and interleukin‐6 (IL‐6) and CRP levels in patients with metabolic syndrome (DiBella et al. 2020) and also that a diet with high choline intake can reduce the risk of developing non‐alcoholic fatty liver disease (Chai et al. 2023). It has been reported that low choline levels may be related to the development of dementia and Alzheimer's disease (Yuan et al. 2022), but, although in animal models the role of choline in delaying cognitive impairment appears to be well established (Gamiz and Gallo 2021; Dave et al. 2023), further studies are needed to support the same role in humans. After its intake, choline is metabolized and can generate metabolites that can also have an impact on health, such as cytidine diphosphate‐choline, which is reported to have a positive impact on mouse models of inflammatory bowel disease, reducing side effects, lowering TNF‐α and IL‐6 levels, and improving gut microbiome composition (Guo et al. 2023). On the other hand, the accumulation of other choline metabolites, such as trimethylamine, which, after absorption by the host, is converted into trimethylamine‐N‐oxide, is being studied for possible negative effects on the development of CVDs and chronic kidney disease (Arias et al. 2020).

### 3.6 Minerals

A good dietary intake of minerals is essential for regulating cell metabolism, crucial biological processes, and biochemical reactions, for maintaining body functions, such as water balance, maintenance of bone integrity, nerve and muscle functions, and for antioxidant and immune responses (Weyh et al. 2022). Low blood levels of minerals and trace elements have been correlated with a higher risk of frailty in older adults (Vural et al. 2020), whereas specific deficiencies of zinc, magnesium, copper, and iron have been associated with depressive disorders and fatigue (Rehault‐Godbert et al. 2019). Eggs are a good source of minerals, such as calcium, phosphorus, and potassium, and essential trace elements, such as selenium, zinc, iron, copper, and magnesium (Table 2); thus, egg consumers have significantly higher levels of intake of calcium, magnesium, and potassium compared to non‐egg consumers (Papanikolaou and Fulgoni 2021). The egg concentration of some of these trace elements can vary greatly depending on the hens' diet. Furthermore, it has been documented that hens raised outdoors lay eggs with a higher content of magnesium, calcium, and zinc compared with caged hens (Elnesr et al. 2024).

## 4 Structural and Bromatological Variances Among Egg Varieties Available on the Market

**FIGURE 2:**
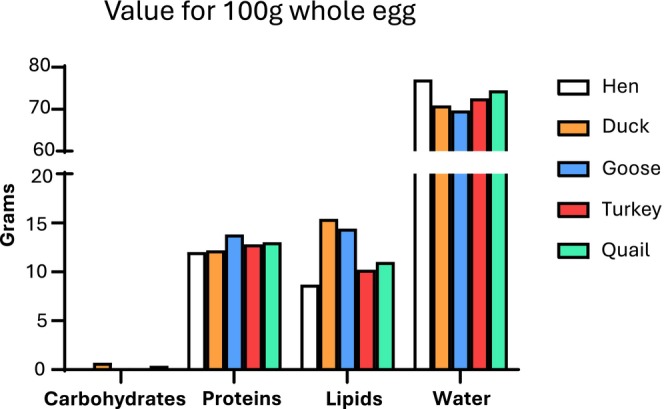
Bromatological composition of different eggs, expressed in g/100 g whole egg.

**FIGURE 3:**
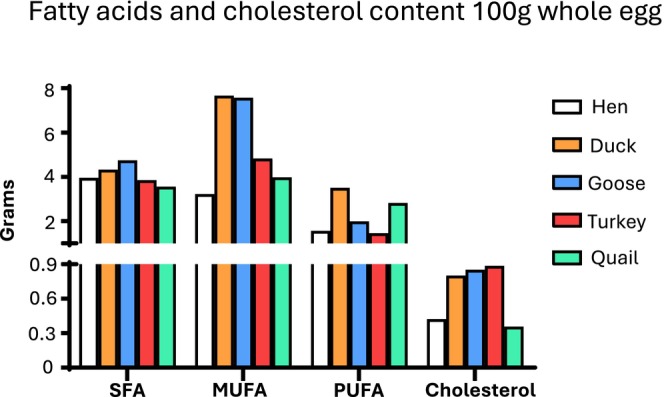
Fatty acids and cholesterol content, expressed in g/100 g whole egg.

Hen's eggs dominate the table egg market worldwide, thanks to several factors, including ease of rearing and annual egg production. Indeed, other laying animal species, such as ducks, turkeys and geese, are defined as seasonal and require extremely precise farming conditions. In addition, the size of the hen egg is considered to be just right, neither too big nor too small. (Rehault‐Godbert et al. 2019). Eggs from different species differ not only in weight and shape, but also to in the proportion of contained yolk. Regarding the weight, goose egg is the heaviest (140 g/egg), whereas quail egg is the smallest (11 g/egg). The proportion of yolk may vary from a maximum of 37.9% for goose to a minimum of 27.5% for hen (Table 3) (Sun et al. 2019). These structural differences are reflected in variations in the bromatological composition and thus caloric value of the eggs themselves (Figure 2). Calories can vary between 128 and 190 per 100 g. Hen egg is the least caloric, whereas duck egg is the most caloric. Concerning protein fraction, the differences are less pronounced with an average content around 12–13 g per 100 g of all types of eggs, with the exception of goose eggs, with a slightly higher protein content (almost 14 g). Regarding lipids, for every 100 g of whole egg, the duck's egg contains the highest amount (15.4 g), followed by goose (14.4 g), quail (11.09 g), turkey (10.2 g) and hen (8.7 g) (CREA, n.d.). Considering the percentage of SFA, the hen's egg shows the highest concentration (45.5%), whereas the duck's egg shows the lowest (28.1%). Duck and quail eggs contain the higher concentration of PUFA (> 22%) compared to other eggs (hen 18.1%, goose 13.8%, turkey 14.3%, quail 11.9%). Goose eggs display the highest concentration of MUFA (52.6%) in comparison with the other species (turkey 47.4%, quail 35,9%, hen 37.1%, duck 22.8%) (CREA, n.d.; Dalle Zotte et al. 2019). Cholesterol content can also vary greatly between species (Figure 3) (USDA National Nutrient Database for Standard Reference, Legacy Release 2019). Mineral and trace element content of hen's eggs is generally lower than the one of other poultry species, especially with regard to calcium, iron, selenium and zinc content, possibly due to the different composition of the birds' diets (Table 4) (Gnagnarella et al. 2022; USDA National Nutrient Database for Standard Reference, Legacy Release 2019).

**TABLE 3:** Egg characteristic of five poultry species adapted from Sun et al. (2019).

	Hen	Duck	Goose	Turkey	Quail
Egg weight (g)	57.75 ± 2.77	74.28 ± 4.67	139.37 ± 10.51	90.44 ± 9.40	11.01 ± 0.77
Yolk weight (g)	15.90 ± 1.27	24.06 ± 1.80	52.79 ± 4.88	26.93 ± 3.37	3.32 ± 0.34
White weight (g)	36.23 ± 1.80	42.79 ± 3.00	72.55 ± 6.68	55.19 ± 6.67	6.71 ± 0.56
Eggshell weight (g)	5.63 ± 0.46	7.43 ± 0.57	14.03 ± 1.63	8.31 ± 0.98	0.98 ± 0.10
Yolk percentage	27.52 ± 1.56	32.40 ± 1.44	37.91 ± 2.62	29.83 ± 2.79	30.19 ± 2.44
White Percentage	62.74 ± 1.49	57.60 ± 1.56	52.03 ± 2.29	60.98 ± 2.81	60.95 ± 2.49
Eggshell Percentage	9.74 ± 0.59	10.01 ± 0.52	10.06 ± 0.85	9.19 ± 0.46	8.86 ± 0.65
Yolk/White	43.93 ± 3.57	56.35 ± 4.02	73.19 ± 8.23	49.22 ± 6.67	49.74 ± 5.49

*Note:* Values are expressed as means ± SD.

**TABLE 4:** Principal minerals and trace elements present in eggs of different species according to Banca Dati di Composizione degli Alimenti per Studi Epidemiologici in Italia (BDA) and department of agriculture, agricultural research service.

	Hen	Duck	Goose	Turkey	Quail
Calcium	48	57	52	50	64
Iron	1.5	2.8	2.6	2.5	3.65
Magnesium	13	16	16	13	13
Phosphorous	210	195	200	200	226
Potassium	133	129	210	142	132
Sodium	137	122	138	151	141
Selenium	0.0058	0.036	0.037	0.034	0.032
Zinc	1.2	1.4	1.3	1.6	1.47

*Note:* Content is expressed as mg/100 g whole egg.

## 5 Recommendations on Egg Consumption at Various Stages of Life

For eggs, the serving size recommended by the Reference Intake Levels of Nutrients and Energy for the Italian Population V Revision 2024 is equivalent to 1 egg (SINU 2024) with a frequency of consumption of 2–4 portions per week according to CREA's guidance ((CREA) CdRAN 2018). However, international public health associations do not define a precise limit to the maximum number of eggs consumed. The Healthy Eating Guidelines of the Agricultural Research Council and Agricultural Economics Analysis, Food and Nutrition Research Center of 2018 stated that eggs, being a food high in nutrient density and relatively high in fat, but with less than 2 g of saturated fat in a unit, should not be harshly condemned since, as previously stated, there is no correlation between the consumption of this food and the incidence of CVDs or increased blood cholesterol levels. Instead, eggs have multiple positive nutritional effects, including a low caloric intake and an excellent protein supply of high‐quality proteins that can promote satiety. Moreover, they are a very practical and versatile food and economically viable due to their low cost. According to the WHO Guidelines for complementary feeding of infants and young children 6–23 months of age, foods of animal origin, including meat, fish, or eggs, should be consumed daily (World Health Organization 2023). The standard recommendations of the European Union on infant and young child feeding up to 3 years of age suggest delaying egg intake to 12 months only in cases of a positive family history of egg allergy, introducing the yolk first and then the white. A 2015 randomized controlled trial conducted in Ecuador showed that the early introduction of one egg per day in children (6–9 months) significantly improves growth, and, being a food accessible to groups of vulnerable populations, egg consumption may play an important role in achieving the overall goal of reducing child stunting due to chronic or recurrent malnutrition (Iannotti et al. 2017). Two studies have highlighted how regular and long‐term consumption of eggs in children can have positive effects on growth, promoting bone health and improving some biomarkers without having negative effects on blood cholesterol. Furthermore, it appears to promote intestinal microbial diversity, maintaining a composition of the intestinal microbiota that benefits health (Suta, Surawit, et al. 2023; Suta, Ophakas, et al. 2023). There is no evidence regarding the number of eggs to consume weekly in the elderly, but being a source of high‐quality protein, easy to consume and economical, they can represent an excellent alternative to other protein sources of animal origin, especially in the elderly, where inadequate protein intake can cause increased skin fragility, a decrease in the immune response, and a consequent poor healing capacity and longer recovery from diseases (Chernoff 2004).

## 6 The Functional Properties of Eggs in Food Technology

Eggs, in addition to their direct consumption, are used in numerous industrial, artisanal, and homemade food preparations and in the production of processed foods thanks to their functional–technological properties, which include excellent emulsification, foaming capacity, and water‐binding and gelling properties (Razi et al. 2023). The protein and lipid fractions naturally found in eggs are exploited and manipulated to exploit these functional attributes.

### 6.1 Emulsifying Property

The emulsification process in eggs is attributed to the protein component of egg whites and the phospholipid content of egg yolks. The EWPs consist of a combination of globular proteins with hydrophilic and hydrophobic regions on their surface, allowing them to form biopolymers with surface‐active properties. This enables them to adsorb at the interface between oil and water, preventing droplet aggregation by creating a surface film and producing electrostatic and steric repulsion effects (Wang et al. 2019). Proteins' emulsifying ability is influenced by their surface hydrophobicity and net charge and the solubility of proteins at the oil–water interface is affected by their surface charge (Chang et al. 2016; Gu et al. 2017). Although EWPs typically exhibit poor emulsifying ability and strong hydrophilic properties, ovalbumin has shown higher emulsifying ability at pH = 3 due to its flexibility and surface hydrophobicity (Wang et al. 2019; Weijers et al. 2006). In acidic conditions, ovalbumin's surface becomes more hydrophobic and flexible, which lowers the kinetic barrier for it to adsorb to the interface, leading to the formation of more stable emulsions (Mine et al. 1991; Xu et al. 2020). The presence of ovotransferrin disulfide bonds inhibits the formation of a transparent cold gel in commercial egg white powder under acidic conditions. Ovotransferrin can stabilize emulsions within specific ranges of pH (Mohseni et al. 2023; Mach et al. 2020; McNamara 2015; Kim and Campbell 2018; Lichtenstein et al. 2021; FAO 2008) and ionic strength (0–1000 mm) (He et al. 2021). Polysaccharides, such as pectin, form a dense interface layer around the droplets that hinder protein aggregation and flocculation, providing strong stability and increasing their viscosity (Alavi and Chen 2022). Affecting the electrostatic interactions between biopolymers, pH plays a crucial role in the formation of protein‐polysaccharide complexes (Xiong et al. 2020). The addition of salts like NaCl can affect the interaction between proteins and polysaccharides in emulsion systems (Souza et al. 2018). Conjugates of proteins and polysaccharides formed through covalent bonding or electrostatic interactions can enhance the emulsifying properties (Dickinson 2009). Moreover, heat treatments can unfold and aggregate proteins, exposing reactive groups that promote interactions between molecules, leading to aggregation and coalescence of proteins. Recently, Chang et al. (2016) described that exposing EWPs to moderate heat (60°C for about 160 min) and acid conditions results in an increase in proteins' net charge and hydrophobicity due to the transition of their third structure and slight modifications in their secondary structure. The type and concentration of the emulsifier used also clearly influence the stability of the emulsion (Razi et al. 2020). Egg yolk phospholipids show high affinity for adsorption at the oil–water interface, making eggs suitable as an emulsifying agent. Various factors, including pressure, temperature, mixing speed, time, droplet viscosity, size, and uniformity, are crucial in maintaining emulsion stability (Anton 2013; Huang and Ahn 2019). Achieving smaller, uniform droplets enhances emulsion quality, mouthfeel, and texture of the final product (Huang and Ahn 2019). The emulsifying capacity of the yolk is used in the food industry for the preparation of finished products, such as mayonnaise, ice cream, and several types of sauces, including salad dressing.

### 6.2 Foaming Capacity and Water‐Binding Activity

Foam is a colloidal dispersion in which a gaseous phase is dispersed in a liquid phase that generally contains a solid part. The technological process for the creation of foam is divided into two steps: production of a gaseous structure, which is incorporated into the product; consolidation and maintenance of the structure obtained. Generally, the number of gas (air) bubbles determines the structure of the product, whereas their size strongly influences its stability (Asokapandian et al. 2015). EWPs are renowned for being excellent foaming agents in the food industry. In the process of whipping the egg white by mechanical treatment, proteins are oriented with the hydrophobic part towards the air phase and the hydrophilic phase towards the aqueous phase. This allows proteins to partially relax, decreasing superficial tension. The balance of the foam thus formed depends on the stability of the film protein, its permeability to air, the quantity of proteins that adsorb to it, and their ability to interact with each other (Mine 2002). Whipping egg whites is a simple operation, but it can be influenced by some factors. Aging of eggs is the first parameter because, as time passes, the yolk and the white acquire a rather alkaline pH determined by the notable loss of carbon dioxide. The yolk goes from a pH = 6 (just laid) to a pH = 6.6; the albumen goes from a pH = 7.6–8.5 (just laid) to a pH = 9.2 after 3 days up to a maximum of 9.7. Furthermore, as the egg ages, ovalbumin becomes less hydrophobic, decreasing the stability of the foam (Alleoni and Antunes 2004). The addition of acids, such as citric, tartaric, and acetic acid, promotes whipping as they allow the negatively charged proteins to come closer together. Furthermore, citric acid whitens the foam as it is capable of binding to the metal ions present inside, which could change the color (Marušić Radovčić et al. 2021). Foams produced at room temperature are faster and have a higher volume compared to those made at colder temperatures because the denaturing activity of proteins takes place more rapidly (John and Flor 1930). Heat treatment, such as pasteurization, can denature EWPs, leading to decreased solubility and increased surface hydrophobicity (Chang et al. 2020). Water can be added with the aim of increasing the volume and obtaining a less dense foam, while the addition of sugar or salt can affect the formation and stability of foam (Razi et al. 2019). Finally, the addition of yolk and fatty compounds in the industrial processing significantly decreases the final volume of foam and its foaming properties (Li et al. 2021). Cold storage (4°C) decreases significantly egg's ability to foam (Chen et al. 2019). Many food products, such as bread, biscuits, cakes, meringues, ice cream, and chocolate mousse, are made using a foam structure, and EWPs play a crucial role in the preparation process.

### 6.3 Gelling Property

Coagulation is a molecular transformation process capable of converting the liquid state of the egg into a gelled, solid state. This major change in structure can occur in both yolk and egg white when, due to a high amount of heat, proteins lose their solubility and transform into a gel (Mine 2002). In the gelled forms, polymeric networks and aggregate dispersions were identified, consisting of globular proteins that have been denatured by heat. With thermal exposure, the intermolecular bonds break, causing the proteins to open with a notable change in the secondary structure from the alpha‐helical form to the beta‐sheet form. To activate the coagulation process, it is important to have a certain degree of opening of the proteins correlated to a partial release of the hydrophobic groups. Proteins are subject to coagulation because of physical agents (mechanical action, temperature) and chemical agents (pH, inorganic ions, heavy metals, ethanol, strong alkali). pH affects protein denaturation, protein‐water interactions, protein–protein interactions, and the overall structure of the gel network. A pH around the isoelectric point results in weaker gels with lower water holding capacity, whereas higher pH leads to stronger gels with better viscoelastic properties (Khemakhem et al. 2019). High pressure treatment affects the gelation of EWPs differently than thermal methods, with a higher ratio of beta‐sheet to alpha‐helical structures observed in thermally denatured ovalbumin. The penetration of water into the molecular cavities is a key factor in pressure‐induced denaturation, leading to a unique array of molecular conformations. Gels formed under high pressure conditions are reported to be less firm but more elastic compared to those formed by heat treatment (Ngarize et al. 2005). The gel formation of EWPs plays a crucial role in the production of a variety of food items, including pudding, desserts, meat products, surimi, and tofu.

## 7 Sustainability of Egg Consumption

Food choices inextricably link people, animals and planet Earth. One of the main concerns about sustainability is related to the impact of the food systems on environmental health, mostly in terms of water, land use and emission of Greenhouse gas (GHG). In the last decade many efforts have been accomplished to analyze the environmental footprint of the agri‐food systems in order to create win‐win dietary patterns meeting dietary needs and environmental health, without neglecting economic, social and cultural issues. This concept is pivotal in the definition of Sustainable Healthy Diets provided by Food and Agriculture Organization (FAO) and World Health Organization (WHO) in 2019. In this framework, the EAT‐*Lancet* Commission conceived the “EAT‐*Lancet* planetary health diet”, a concrete model of Sustainable Healthy Diet which could respect the safe operating space defined for agri‐food systems to preserve our planet resources (Willett et al. 2019). Globally, egg consumption is higher than the suggested intake of 13 g/day, proposed by the EAT‐*Lancet* Commission (Willett et al. 2019), and even the suggested intake could be restricted due to sustainability reasons linked to the environmental footprint of their production. Poultry production systems are different depending on farming methods and production purposes (Gerber et al. 2013). Backyard layer productions consist in small‐scale poultry systems kept by households for personal consumption of meat and eggs or for sale at local scale of small quantities. On the contrary, industrial layers and broilers refer to large‐scale commercial poultry systems producing copious quantities of eggs and meat for the broader market (Augère‐Granier 2019). There are also different housing types: conventional cage, enriched colony and cage‐free housing (barn, aviary, free‐range, organic housing) (Majewski et al. 2024; Ochs et al. 2018). According to the Council Directive 99/74/EC and the related regulations on layers housing and commercialization, from 2012 the European Union market admit only eggs coming from EU‐compliant enriched cage systems or cage‐free systems (Union 1999; Molnár and Szőllősi 2020). Egg production sustainability depends on many factors within the three dimensions of Sustainable Development—environmental, economic and social—enlightened in The 2030 Agenda for Sustainable Development, a resolution adopted by the United Nations General Assembly (Nations U. Department of Economic and Social Affairs Sustainable Development 2024). The environmental footprint evaluation is usually estimated with the Life Cycle Assessment (LCA) approach and focuses on feed production and manure management, since livestock represents the major emission source of the sector. (Maciel et al. 2023; Leinonen and Kyriazakis 2016). According to FAO evaluation, chicken supply chains are responsible for the emission of 606 million tones CO_2_‐eq, accounting for the 8% of livestock sector's GHG emissions (FAO (Food and Agriculture Organization of the United Nations), n.d.). Feed production is the main driver of GHG emissions for egg supply chain, representing around 57% of the total emissions. Feed composition and output management are different speaking about egg or broiler industries. On the one hand, broiler feed needs a higher percentage of protein mostly achieved with soybeans encouraging land‐use conversion. On the other hand, manure management is less threatening in broiler industry because it's managed in dry aerobic conditions lowering the emissions around 6%, layers manure instead is stored in long‐term pits in liquid conditions reaching 20% of total production emissions (Gerber et al. 2013). Backyard poultry can be considered as a more sustainable alternative, since a flock of 5 to 20 could be easily managed by a family without professional feeding and housing facilities reducing environmental impact due to manure management and feed production (Animal Production and Health Division 2014). Furthermore, this method enhances biodiversity conservation and could be integrated with other agricultural operations thanks to the manure rich in soil nutrients (Singh et al. 2022). However, productivity and quality are key economically speaking and industrial systems are way more productive compared to backyard systems. Furthermore, egg production costs are higher in enriched‐cages and non‐cages systems due to a higher feed consumption, higher mortality, higher cannibalism occurrences and lower stocking density. Overall, conventional cage systems seem to be the most effective in terms of costs and productivity. However, in the last few decades consumer demand of organic products has become a market trend, influenced by the common bias that organic production may necessarily correspond to high‐quality products and to healthier choice. The animal welfare in intensive productions is increasingly sensitizing consumers, as a result a social concern regarding egg production about animal and human health is widespread (Maciel et al. 2023). Moreover, the backyard poultry systems are linked to social empowerment of women, since they are the one managing the household facilities, and their business could be considered as an example of circular economy converting kitchen waste and soil resources into a commodity (Alders et al. 2018; FAO and IFAD 2022). Furthermore, eggs represent a cost‐efficient commodity, since they are source of protein, choline, bioactive compounds and some important micro‐nutrient, and the estimated cost is low, playing a crucial role in food security (Papanikolaou and Fulgoni 2020). Although the concern about the sustainability of our diets is necessary, the consequences of replacing animal source foods with plant‐based source foods must be carefully considered. Since the amounts and the bioavailability of some micronutrients from animal sources are higher compared to plant‐based sources, such as vitamin B12, iron, calcium and zinc, the micronutrient adequacy must be extensively evaluated. In this regard, several critical aspects of “The EAT‐Lancet planetary health diet” were pointed out. This dietary pattern was assessed to be inadequate in terms of micronutrients due to a low content of animal protein sources and a high content of phytates which reduce the bioavailability of some micronutrients (Beal et al. 2023). Diversifying animal protein sources and preferring those with a lower environmental impact is key to move towards more sustainable dietary patterns. Regardless the farming method, poultry products represent an excellent compromise, having a low global average emission intensity, below 50 kg CO_2_‐eq per kg of edible protein for chicken meat and eggs, which is extremely lower compared to beef meat and quite similar to cattle milk (respectively over 300 and less than 100 kg CO_2_‐eq per kg of edible protein) (Augère‐Granier 2019). Moreover, they have a relatively low water footprint (estimated 2562 L of water/kg) compared to other animal‐sourced protein food, such as bone‐free meat, and cow milk (estimated 15,139 L and 1260, 5 L of water/kg, respectively) (Petersson et al. 2021). Although plant‐based source foods have a lower environmental footprint compared to animal source foods, it is necessary to point out that current indicators based on kg of food commodity penalize nutrient‐rich foods, such as protein‐rich foods, and therefore the use of indicators like the nutrient LCA, which also take into account nutrient content, is essential (McLaren et al. 2021). Furthermore, even if the backyard poultry systems seem to be more sustainable than the industrial systems in terms of environmental impact, economic and social dimensions of sustainability must be taken into account since their productivity is lower and would not be able to meet the demand of the growing global population. Therefore, coming to a compromise is necessary, which may include avoiding over and under‐consumption of eggs and considering backyard poultry as a valuable alternative to industrial systems in lower‐income developing countries (FAO and IFAD 2022).

## 8 Conclusions

Given the recent studies that demonstrate a lack of correlation between cholesterol and CVDs (Formisano et al. 2025), and given the beneficial properties of various nutrients it contains, this review shows that egg is a safe food that provides energy, important nutrients, and has several chemical–physical properties that make it particularly useful in the preparation of various recipes and in the food industry. Within various dietary models, including vegetarian, flexitarian, Mediterranean, and DASH, eggs find an adequate space, also compatible with the concept of sustainability, especially when the producer respects the animal welfare policy.

## Author Contributions

**Irene Caffa:** conceptualization (equal), supervision (equal), writing – original draft (lead), writing – review and editing (equal). **Elisa Proietti:** conceptualization (supporting), writing – original draft (equal), writing – review and editing (equal). **Federica Turrini:** conceptualization (supporting), writing – original draft (equal), writing – review and editing (equal). **Consuelo Borgarelli:** conceptualization (supporting), writing – original draft (equal). **Maria Regina Ferrando:** conceptualization (supporting), writing – original draft (equal), writing – review and editing (equal). **Elena Formisano:** writing – review and editing (equal). **Lenycia de Cassya Lopes Neri:** writing – review and editing (equal). **Daniela Martini:** writing – review and editing (supporting). **Donato Angelino:** writing – review and editing (supporting). **Anna Tagliabue:** supervision (equal), writing – review and editing (equal). **Livia Pisciotta:** conceptualization (lead), supervision (lead), writing – review and editing (equal).

## Conflicts of Interest

The authors declare no conflicts of interest.

## Data Availability

Data sharing not applicable to this article as no datasets were generated or analyzed during the current study.
